# What’s in a game? A systems approach to enhancing performance analysis in football

**DOI:** 10.1371/journal.pone.0172565

**Published:** 2017-02-17

**Authors:** Scott McLean, Paul M. Salmon, Adam D. Gorman, Gemma J. M. Read, Colin Solomon

**Affiliations:** 1 School of Health and Sport Sciences, University of the Sunshine Coast, Sippy Downs, Australia; 2 Centre for Human Factors and Sociotechnical Systems, University of the Sunshine Coast, Sippy Downs, Australia; Universidade de Tras-os-Montes e Alto Douro, PORTUGAL

## Abstract

**Purpose:**

Performance analysis (PA) in football is considered to be an integral component of understanding the requirements for optimal performance. Despite vast amounts of research in this area key gaps remain, including what comprises PA in football, and methods to minimise research-practitioner gaps. The aim of this study was to develop a model of the football match system in order to better describe and understand the components of football performance. Such a model could inform the design of new PA methods.

**Method:**

Eight elite level football Subject Method Experts (SME’s) participated in two workshops to develop a systems model of the football match system. The model was developed using a first-of-its-kind application of Cognitive Work Analysis (CWA) in football. CWA has been used in many other non-sporting domains to analyse and understand complex systems.

**Result:**

Using CWA, a model of the football match ‘system’ was developed. The model enabled identification of several PA measures not currently utilised, including communication between team members, adaptability of teams, playing at the appropriate tempo, as well as attacking and defending related measures.

**Conclusion:**

The results indicate that football is characteristic of a complex sociotechnical system, and revealed potential new and unique PA measures regarded as important by SME’s, yet not currently measured. Importantly, these results have identified a gap between the current PA research and the information that is meaningful to football coaches and practitioners.

## Introduction

Since the 1960s, football researchers have investigated the physiological, technical, and tactical components of football to determine the key performance indicators (KPIs) that predict successful performance [1, 2]. In more recent times, advances in computer and video aided match analysis systems, as well as increased global visibility and reach, has led to a substantial increase in football performance analysis (PA) literature and methods [1, 3, 4]. Despite more than five decades of research in this area, current football PA methods remain beset by various issues, including a lack of standardised operational definitions, a lack of match context, and the discrete measurement of isolated variables [1, 4]. Furthermore, previous PA research has had only a minimal impact on practice [5, 6], suggesting a lack of transferability of research outputs to practice [1, 5]. One reason for this is that football match performance has not yet been described in its entirety. Accordingly, there remains a substantial number of features that need to be defined and measured in football PA to ensure that the data are of benefit to practitioners [1, 5].

This is perhaps not surprising, as football matches possess many of the characteristics of complex sociotechnical systems [7, 8]. That is, there are multiple interacting human and non-human components operating within a dynamic and constantly changing match environment. A corollary of this is that football performance is highly complex, multi-faceted, and ultimately difficult to define. Football performance is more than the sum of its parts. Given this, the extent to which football performance and the factors influencing it are fully understood is questionable [5]. Existing approaches to performance analysis can be thought of as reductionist. They rely on taking the system apart in order to understand the components (e.g., players, passes), then assess the performance of those components in isolation before reassembling them back into the complete system, on the tacit assumption that the whole simply represents the sum of its parts [1]. Reductionist approaches do not allow the development of a complete understanding of performance and the factors influencing it, nor do they allow the detection of new emergent behaviours that could augment performance [9]. For PA to provide valid assessments of football performance, it is argued in this study that new methods for PA may be needed, particularly given that the current approaches to PA have changed very little over the past 25 years (see review by Mackenzie & Cushion, 2013[1]). Although, positive developments regarding player and team movement patterns are beginning to emerge as new technology becomes available, there is work to be done in integrating the measures appropriately. For example, research grounded in ecological dynamics theory allows for some understanding of non-linear dynamics of performance, by combining traditional notational analysis with spatio-temporal analysis [10, 11]. Investigations into attacker-defender dyads, and the effects of match constraints (opposition, team members, the ball, goalposts, etc.) on the emergent coordination patterns during team sports, have advanced traditional PA methods [10, 12].

An alternative approach to understanding performance in football is offered by some of the methods being applied within the discipline of Human Factors (HF). Human Factors researchers study the performance of humans in sociotechnical systems, and in recent times there has been a shift towards “systems thinking” methods that are used to describe and assess overall complex system performance [13]. Traditionally, HF methods have been applied to complex systems, such as road safety, the military, and aviation domains, to provide safer and more efficient and effective systems. For example, in road safety attention has shifted from identifying the driver-related behaviours that cause road trauma (e.g. speeding, drink driving) toward modelling the entire road system and identifying the system wide conditions that interact and lead to or enable drivers to engage in risky behaviours (e.g. factors related to road safety policy, education, enforcement, training, licensing, and road infrastructure [14]. A key contribution of HF methods is the ability to represent complex systems and the interacting factors that play an important role in determining how the system behaves.

Although typically applied in the safety critical domains, such as road safety and aviation, there is scope for these methods to be applied as part of a multi-disciplinary approach to sport science [15]. Indeed, the high explanatory power of systems approaches is now beginning to be recognised in sports science disciplines [16]. One such method, Cognitive Work Analysis (CWA) [17], has been used to analyse, design, and evaluate complex sociotechnical systems across a diverse range of domains [18–20]. One of the fundamental strengths of the CWA framework is the capacity to describe, in-depth, complex sociotechnical systems and the factors influencing performance [21]. In particular, the first phase of CWA, Work Domain Analysis (WDA), is used to construct an in-depth description of the functional structure of the system under analysis. This functional structure covers the purposes of a particular system, the objects used, the behaviours required for successful performance, and criteria that is used to discriminate between good and bad performance. More specific details of the CWA approach are provided in the Methods section.

The first step in developing new and more appropriate football PA methods involves identifying what ‘performance’ in a football match actually comprises and how the different facets of performance interact to influence and determine match outcomes. That is, by describing in-depth all of the behaviours required for optimal performance, it will be possible to judge, first, whether existing PA methods are comprehensive, and second, if they are not, determine what additional measures are required. Therefore, the aim of this study was to conduct a first-of-its-kind application of the CWA framework that describes a football match. The study was designed to develop a complex systems model of a football match in order to identify the interacting network of components that require measurement for comprehensive PA. This model is then used to assess the current literature and methods for PA for gaps in knowledge and methods.

## Methods

### Study design

**Fig 1:**
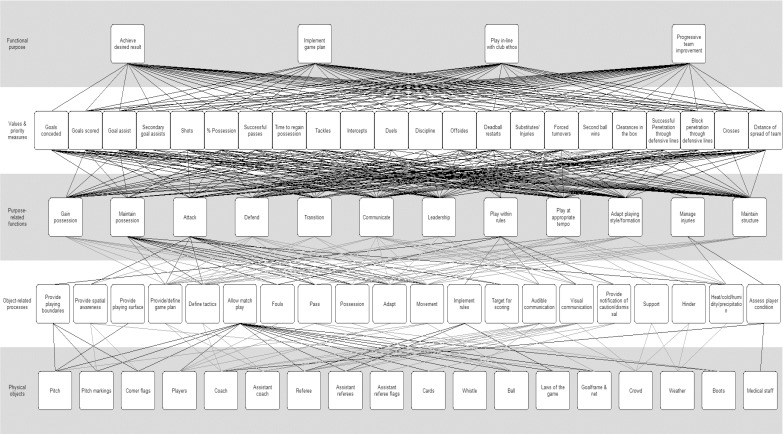
AH of the football match system.

The current study used the first phase of CWA, WDA, to develop a model of the football system to describe in detail what comprises football performance. Initially, the research team developed a draft model of the football match system, within the predetermined analysis boundaries. The research team comprised one football expert (former player, current coach, and football researcher), two HF practitioners with extensive experience in applying CWA in systems analysis and design [19], and two sport scientists. Two subject matter experts (SME’s) workshops were then conducted to review and refine the model. The workshop process involved familiarising participants with the CWA framework and associated methodologies, presenting a prototype analysis developed by the research team, and then subsequently working through the analysis with the SME’s until consensus was achieved. In the workshops, the SME’s were given an introduction to the WDA framework and the research project aims, before reviewing and refining the draft model in an open and guided group discussion. The workshops were structured so that each of the five levels of abstraction, the means-end links (see below for definitions), and the terms within the nodes (concepts related to the appropriate level of abstraction), were reviewed and refined by the SME’s. The SME’s were questioned on the appropriateness of the nodes, whether items needed to be included or excluded from the model, if the linkages between nodes were appropriate, and if the terms were understandable for practitioners and coaches. The SME’s were also questioned on how the model could be used to generate new measures of performance. The model was progressively refined during workshop discussions, and the SME’s workshops were voice recorded, which was subsequently reviewed and used to assist with the production of the current model of the “football system” (Fig 1).

### Cognitive Work Analysis (CWA)

Cognitive Work Analysis (CWA) [17] is a systems analysis and design framework that has previously been used both to analyse complex sociotechnical systems and to inform system design or redesign activities [22, 23]. A fundamental feature of the framework is that it is used to determine the constraints imposed on behaviour within the system [21]. For example, a footballer’s movement on the pitch is constrained by the line markings, the physical presence of other players as well as the rules of the game and the tactics set by the coach. Understanding the constraints enables resulting design recommendations to explore the potential benefits of making constraints more explicit to users, removing constraints on behaviour, or better exploiting existing constraints to support behaviour. The framework comprises five phases; however, the number of phases used is dependent on the purpose of the analysis. This study uses the first phase of CWA [17], ‘Work Domain Analysis’ (WDA) to model the football match system.

### Work Domain Analysis (WDA)

WDA is used to provide an event and actor independent description of the system under analysis: in this case an elite football match system. The aim of the WDA is to describe the purposes of the system, the objects within the system (both human and non-human), the functions performed, the inter-relations between components, and the constraints imposed on the actions of any actor (e.g., player) performing activities within that system [17]. This is achieved by describing systems at five conceptual levels using the abstraction hierarchy (AH) method as follows:

1. Functional Purpose–The overall purposes of the system and the external constraints imposed on its operation. For example, in a football match, these would include to achieve the desired result and/or to implement the game plan;
2. Values and Priority Measures–The criteria that are used for measuring progress towards the functional purposes. For example, in relation to football, this level would include values and measures that a football team uses to determine whether or not it is achieving the functional purposes of a particular match. Example values and measures would include the match score, amount of possession, and attempts on goal;
3. Generalised Functions–The general functions of the system that are necessary for achieving the functional purposes. For example, to attack, defend, transition, and maintain possession.
4. Physical Functions–The functional capabilities and limitations of the physical objects within the system that enable the generalised functions. For example, a player can kick and head the ball, run, communicate, and tackle; and
5. Physical Objects–The physical objects within the system that are used to undertake the generalised functions. For example the players, the ball, pitch, crowd, referees, and coach.

The output is a detailed description of the football match system, in terms of the constraints influencing behaviour and how the physical objects and the functions they support enable the system to achieve its functional purpose. Importantly, the abstraction hierarchy model uses means-ends relationships to link nodes across the five levels of abstraction. The means-end links show the why-what-how relationship between each of the nodes at different levels [24]. For example, in the context of a football match, if the what is ‘attack’, the why above it could be to ‘score goals’ and the how below it could include capabilities such as ‘pass’, ‘possession’, ‘move’. This is an important feature of the analysis as it moves from a reductionist component perspective to show the relationships between different aspects of performance.

### Analysis boundaries

As stated, the aim of the present analysis was to conduct a systems analysis of football match performance. This was achieved through applying the first phase of CWA, WDA. Specifically this involved developing an Abstraction Hierarchy (AH) model of football match performance. The focus of the analysis was confined to the 90 minutes of an elite level match. This meant that factors related to performance that occur outside of the match, such as training and nutrition, were not considered. A second aspect of the boundaries was related to the physical and physiological components of performance, such as heart rate, and energy expenditure etc., which were not considered in the analysis as they do not indicate successful or unsuccessful match performance.

A third and final boundary placed around the analysis was a focus on professional level football. Professional football provides the most representative data from which to determine the types of tactics and skill related variables that are employed by expert players. The examination of expert behaviours provides important evidence that can be used as a benchmark for further investigations aimed at examining whether similar trends emerge in lesser skilled competitions.

### Subject matter experts (SME’s)

Institutional ethical approval was granted for the study. Approval number S/16/913. The Human Research Ethics Committee of the University of the Sunshine Coast granted expedited ethics approval for the project. Accepting the workshop invitation, and attendance at the workshops was constituted consent to participate in the project. The process involved the research team liaising with SME’s to construct the AH. Twelve SME’s were identified by the research group as experts in elite level football, and invited to attend one of two workshops. In total eight male SME’s accepted the invitation and attended the workshops. The number of SME’s in other studies using CWA methods ranges from three to eleven [20, 25, 26], therefore eight SME’s is well within the range that is considered appropriate for conducting similar analyses, especially given that consensus on the model was achieved. The relevant football SME’s had extensive playing, and coaching experience across Europe, Asia, and Australia (Table 1). The SME’s nationalities were English, Scottish, Welsh, and Australian.

**Table 1:** Subject matter expert (*n* = 8) experience.

Description	Subject Matter Experts								
	P1	P2	P3	P4	P5	P6	P7	P8	Total
Professional/semi-professional coach (ys)	5	-	3	25	41	15	4	-	93
International matches coached	-	-	15	50	48	-	-	-	113
Professional matches coached	60	-	30	-	138	-	-	-	228
Semi-Professional matches coached	-	20	110	400	800	300	100	-	1700+
Major tournament matches coached	-	-	6	6	-	-	-	-	12
Academy coach (ys)	-	-	2	1	14	-	2	-	19
Current coach accreditation	Pro	B	Pro	A	A	B	A	A	-
International matches played	57	52	5	-	-	-	20	-	134
Professional matches played	459	314	300	117	-	-	-	-	1190
Major tournament matches played	10	40	35	6	-	-	-	-	91
Professional match analyst (ys)	-	-	-	-	9	-	-	2	11

**Note:** Major tournaments include World Cup, Olympics, Youth World Cup, Asian Cup, European Champions League, and European Youth Championships. The number of semi-professional games coached by the SME’s were reported as estimates. The coach accreditation levels were obtained from either the European (UEFA) or Asian (AFC) football confederations.

## Results

### Work domain analysis

The SME’s revised ‘football match system’ AH is presented in Fig 1.

The WDA is discussed below with reference to the current literature on football PA.

#### Functional purposes

At the Functional Purpose level, four Functional Purposes of a football match were identified by the SME’s: (1) achieve desired result, (2) implement game plan, (3) play in line with club ethos, and (4) progressive team improvement (Fig 1). Although the SME’s agreed upon “achieve desired result” as the primary Functional Purpose at an elite level, the three other Functional Purposes identified were discussed and resolved to be sufficiently important to be regarded as additional Functional Purposes. Notably, the SME’s reported that the importance placed on each of the Functional Purposes shifts depending upon game context. For example, in important matches, achieving the desired result would be the most important purpose. Conversely, in matches of lesser importance, even if a team does not achieve the desired result, a coach may still be satisfied if the other Functional Purposes are met such as being able to successfully implement the game plan.

An interesting feature of the functional purposes level is the often conflicting nature of the functional purposes identified. For example, it may be that playing in-line with the club ethos may conflict with the primary functional purpose of achieving the desired result (e.g., when the club ethos is to play attacking football, and yet the most favourable tactic against a given team may be to play more defensively). In relation to PA, there appears to be little in the way of specific measures designed to assess the functional purposes of ‘implement game plan’ and ‘progressive team development’.

#### Values and priority measures

Several of the identified Values and Priority Measures, such as passing success, shots, tackles, and intercepts, have previously been measured to assess performance in football [27]. However, it was emphasised by the SME’s that these measures are only useful when examined in the context of where they occurred on the pitch. The SME’s deemed the inclusion of the area of the pitch, and the ability to present results across pitch areas, to be critical requirements for PA methods.

**Fig 2:**
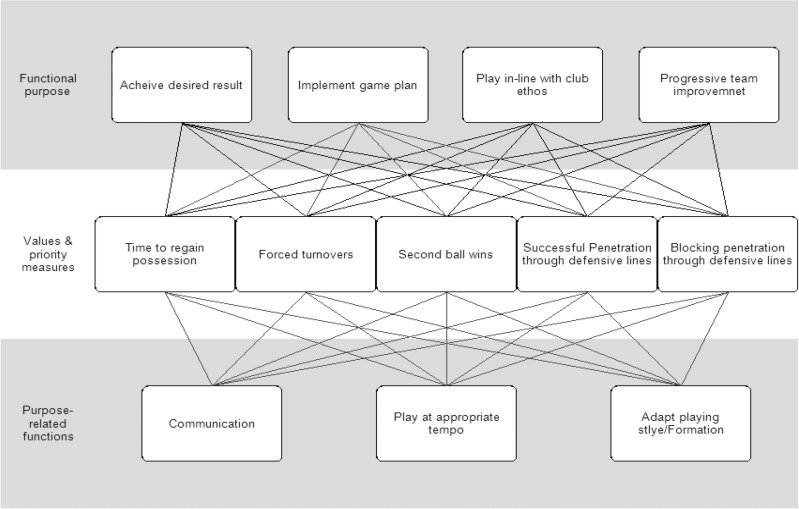
Extract of the AH representing the novel PA measures.

Notable features at the Values and Priority Measures level was the identification of new measures that the SME’s indicated were important, such as playing through defensive lines and blocking penetration of defensive lines, time to regain possession, forced turnovers, and second ball wins (Fig 2).

#### Purpose related functions

Functions identified at this level included the high level functions of attack, defend, gain and maintain possession, and transition from attack to defence and defence to attack. These have all previously been measured using a variety of different methods [28–30]. However, several new functions, described in the current model were deemed important by the SME’s, which included verbal and non-verbal communication between players, playing at the appropriate tempo, and team adaptability (adapting the style of play to suit a particular scenario).

The lower two levels of the AH, Object Related Processes and Physical Objects, show the physical objects in the systems and what they provide or ‘afford’ in terms of contributing to achieving the system’s functions. For example, the pitch provides a playing surface, the goal frame provides a target for scoring, the players afford movement and passes, and the coach provides team tactics and a game plan.

As discussed, a number of the nodes identified in the AH represent features of football performance that are not currently considered by existing PA methods. An indication of the relative importance of these new items can be derived by examining the relationships between nodes as indicated by the means-ends links in Fig 1. For increased clarity (Fig 2) shows the novel measures and their relationship (links) to the Functional Purposes. For the Values and Priority Measures, the links indicate a direct relationship with variables at the level above and below in the AH. The Purpose-Related Functions identified in the AH that are not considered by existing PA methods are shown in (Fig 2), and indicates a relationship with variables at the levels above. As shown by the number of relationships expressed in (Fig 2) the Values and Priority Measures and Purpose-Related Functions not currently measured by PA methods represent important aspects of performance.

## Discussion

The aim of this article was to present a first-of-its-kind WDA of the ‘football match system’ in order to examine the state of the art in football PA. The WDA was developed based on two workshops involving highly experienced football SME’s. The study was designed to determine the composition of high performance football, and to then use this to identify key knowledge gaps within the PA literature.

### Issues identified in current football performance analysis

Three major contributions to PA research were identified in the current study. Firstly, the analysis confirmed that the game of football is indeed characteristic of a complex sociotechnical system. Developed based on input from experienced elite football SME’s, the WDA provides a detailed description of the football system and evidence of the complexity of football. There are multiple components (e.g., the physical objects) that dynamically interact to influence the match outcome (e.g. the means-ends links) (see Fig 1 and Fig 2). In addition, there are multiple processes occurring simultaneously in pursuit of multiple functional purposes. Presenting the game of football as a complex system emphasises the many competing functions and relationships between the individual components during a match. An important implication of the current method over existing methods, is that existing PA methods typically fail to consider this complexity, instead often focussing on components in isolation (e.g. passing) [31] or a limited number of interacting variables.

Secondly, a substantial contribution of the analysis is that it has identified aspects of performance, considered by the elite level football SME’s as important to optimal match performance, that are not currently measured (e.g. adaptability, communications), where existing knowledge is minimal (e.g. tempo, regaining possession), or where the investigated variable is not currently measured in the appropriate context (e.g. area of the pitch where important actions occur).

Thirdly, the WDA revealed a substantial gap that exists between current football PA literature and the measures that are useful to coaches in everyday practice. The SME’s reported that many of the PA measures in the literature are in fact not useful in practice, either because they are too complex (e.g., mathematical based methods), are too reductionist (e.g., passing measures that do not consider pitch areas), or do not incorporate important aspects of football match performance. The remainder of this discussion focuses on these key findings and their implications for PA research.

Despite the rapid and continual advances of football, the analysis presented supports assertions by other researchers that football PA research has failed to keep up [1]. Another issue with PA literature is that too often PA research falls into the interesting but not useable category. For example, research that includes substantial and complex statistics and mathematical equations [32, 33] can make interpreting the implications difficult, and therefore, unlikely to be adopted by coaches [9]. The SME’s agreed that coaches and practitioners prefer straightforward analysis that provides a quick “snapshot” of the team’s performance [9]. However, the SME’s stressed that at the elite level, team performance analysis is preferable to individual performance analysis. Consequently, performance analysts may be investing valuable resources obtaining individual performance data using reductionist methods such as frequencies and percentages that are not the highest priority for coaches. Furthermore, the usefulness of compiling match performance data of individual players into a database to compare to previous performance is questionable when it is known that technical skills and physical variables vary depending upon a number of other variables such as match location, opposition strength, and match outcome [25, 34].

### New PA measures

Based on a comparison with the existing PA literature, the WDA revealed multiple novel football performance Purpose Related Functions, and Values and Priority Measures. Importantly, these levels of the WDA presents a range of potentially new measures of football performance that the SME’s indicated were important (Fig 2). Novel Values and Priority Measures included time to regain possession, forced turnovers, second ball wins, and penetration through defensive lines, as well as blocking penetration through defensive lines (Fig 2). For the Purpose Related Functions, team adaptability, communication, and playing at the appropriate tempo were identified as important to achieving the functional purposes, and are yet not measured or fully understood in the PA literature in football. The WDA indicates that an expansion of the current PA measures is required for research to keep pace with the coaching process and coach’s needs. In addition, it suggests that an integrated set of measures is required, as opposed to the use of isolated individual measures. The following discussion addresses the novel measures identified by the WDA, and propose future directions for football PA research.

#### Areas of the pitch

A pertinent finding was the need for more detailed information regarding the specific locations on the pitch where functions are performed. It is recommended that all of the Values and Priority Measures in the model be analysed by considering pitch locations to provide useful information for coaches. This view is supported by Mackenzie and Cushion (1) who identified that PA research does not always include the context of pitch location in relation to important match events. An example was that the SME’s expressed a desire to know where on the pitch and how their team gains possession, and then where and how the team progresses its attack. This type of analysis can be achieved by dividing the pitch into zones to provide a segmented map of the pitch [35], enabling match analysis within the context of field position. Using this method, Gómez, Gómez-Lopez (35) were able to identify specific zones on the pitch where important actions occurred. For example, they showed that winning teams had better ball retention in attacking areas, resulting in a greater number of shots and goals [35]. However, this simple context is often not reported in PA literature [1].

#### Penetration of defensive lines

**Fig 3:**
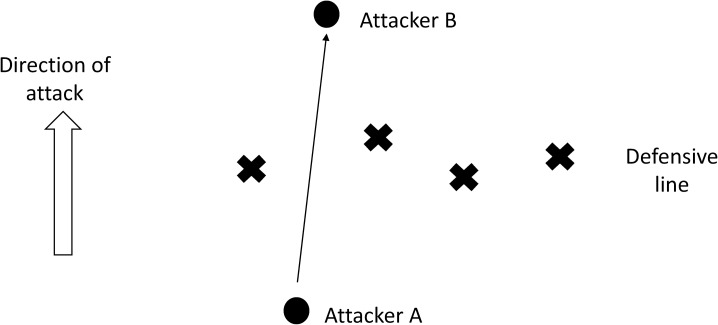
Schematic of a pass penetrating a defensive line.

Penetrating through, and blocking penetration through, defensive lines were included in the WDA as important measures of both attack and defence. However, this has not previously been demonstrated in the literature, despite the apparent importance of these variables to coaches, according to the SME’s. A simple schematic representing a pass through the defensive line is shown in (Fig 3). The important measure that emerges here is the number of players rendered positionally “out of play” by the penetrating pass. The example in (Fig 3) shows that four players in the defensive line are now on the wrong side of the ball in relation to their goal, which could present the opportunity of a numerical imbalance in favour of the attacking side. The opposite of this scenario is where penetration of the defensive line is prevented, thereby allowing the four defensive players to maintain their numerical and positional advantage. These measures are important to coaches, and would have a practical impact, yet are not apparent in the PA literature. This is one example of the research-practitioner gap whereby aspects of performance considered important by coaches are not considered by researcher methods.

#### Gaining possession

The importance of possession in football has been previously reported [36, 37]. Tackles and intercepts are currently measured as indicators of gaining possession [27, 38]. However, these measures are reductionist, as they are often the result of the preceding events and therefore fail to fully capture the contextual factors that led to the event itself. Measuring forced turnovers and second ball wins (described below) would provide a better understanding of gaining possession. A forced turnover involves forcing the “in possession” team into an area where the “out of possession” team is able to apply pressure and win the ball, by either a tackle or interception. This style of defending allows some degree of control over the match even when not in possession. Subsequently, a successful forced turnover could mean possession is gained in a favourable area of the pitch from where an attack can be initiated. Second ball wins refer to instances where the ball is not in the possession of either team and possession is gained. For example, when two players challenge for a header from a long ball and the ball breaks away from both players. The second ball win is achieved by the team who then gains possession of the loose ball. It was apparent from the SME’s that this is an important method of gaining possession, and that some teams’ tactical structure is to position players in areas where they can win second balls to gain possession e.g. in the attacking third. Simply measuring tackles and intercepts are examples of how reductionist measures often only show an outcome, which does not allow a full understanding of the preceding event, which would be of greater value to coaches. For example, a team may lose many one-on-one challenges but may have a significant success rate in winning the resulting second balls. A ‘tackles won’ only assessment would therefore provide a misleading picture of performance.

A further measure identified in the WDA was the importance of knowing the time taken for a given team to regain possession. Only one study has investigated the time to regain possession (termed defensive reaction time) using variables such as team ranking (top, middle, and bottom teams) and match status (winning, drawing, losing) [30]. The top teams had a faster defensive reaction time compared to middle and bottom level teams, and losing teams (during the match) had faster defensive reaction time compared to winning teams [30]. This research confirms the need to include time to regain possession within the current model. However, future analysis could report the location on the pitch of successful possession regains, and also the match to match variability of a team’s defensive reaction time. This additional information would provide coaches with useful information that could benefit the coaching process.

#### Adaptability

Team adaptability was identified in the WDA as a key function influencing performance in football matches. Interestingly, whilst adaptability is well established as a key feature of high performing teams in other domains [39], there is a lack of research relating to adaptability in team sport. It is well known that a critical attribute of elite individuals is their capability to adapt to the continually changing demands within the sporting context [40–42]. Whether this phenomenon relates to team adaptability has received limited attention in the literature and is clearly a topic of importance to understand the influence of adaptability on the outcome of a match. Team adaptability could encompass the ability of teams to adapt tactics and formations to certain situations in a match, across a season, or within a specific tournament. Adaptability may be necessary when the chosen structure or coaching philosophy is not progressing as planned, and needs to be changed. Specific measures of adaptability could arise from the responses to such changes made, for example, assessing whether changes to structure or formation allowed the team occupy into new areas of the pitch that they were previously unable to get into, or whether the changes stopped any problems that were occurring. These are examples of measureable responses of adaptability that could be useful for coaches to understand the effect of the chosen adaptations on specific outcomes. Given the strong evidence that shows how more successful individuals tend to exhibit high levels of movement adaptability [40–42], future football PA should investigate the measurement of team adaptability.

#### Communication

The relationship between team communication and performance in elite football is yet to be established, however, evidence from sporting contexts other than football have highlighted the important role of effective communication in team performance [39, 43]. For example, winning tennis doubles teams exchange messages more frequently, and focus more on action (task) specific statements, compared to losing doubles tennis teams whose communication is mainly non-task oriented [43]. The means-end links in the WDA highlights the importance of communication to achieving the functional purposes, yet there is no known football communication measurement tool. Notably, methods such as social network analysis [44] are increasingly being applied to measure communications in a diverse range of domains. An interesting research direction would be to firstly devise a method to measure communication in football, and then to measure the communication between players during actual matches. It could be useful to measure communication in attack, defence, and in the context of match status. This approach would provide the coach with information regarding the major communicators in attack and defence, and then allow these communications to be investigated in regards to match outcome. The SME’s indicated that when winning, the communications tended to be task specific, however, when losing, team communications tended to be emotional. This information could present valuable opportunities for coaching, for example, players could be coached to focus on task communication when losing, rather than emotional communication.

#### Playing at the appropriate tempo

Playing at the appropriate tempo was identified in the WDA as a prominent function for achieving the desired outcomes during a match. The term tempo is typically used in football to describe the speed of play [45–50]. However, definitions of football tempo are varied. One study defined playing tempo by the number of touches taken in possession, with a low amount of touches per ball possession (1–2) classified as high tempo, and greater than three touches per ball possession as low tempo [48]. Another study reported ball speed (m/s) and passing rate (passes per minute) as indicators of match speed during World Cup finals [49]. Despite this research, the relationship between tempo and successful performance is still unknown. A combination of the above measures, with additional components of speed of movement of the attacking and defending teams, and distance gained in possession, could possibly add to the definition of tempo. This would help to inform football coaches on how teams regulate tempo during a match including, for example, whether teams start fast and finish slow, or whether more goals are scored as a result of a faster tempo. Firstly, tempo must be clearly defined.

As a first-of-its-kind application it is important to note the strengths and weaknesses of using WDA to describe and analyse the elite football match system. A strength of WDA is that it does not attempt to describe or predict the behaviour of the individuals within the analysis, but instead describes the constraints in the system that can influence and effect behaviour. This is important because coaches, clubs, and players each have their philosophies on how football should be played. Coaches may need to consider including the additional variables identified in this study in their current PA approaches to provide a more comprehensive and informative (i.e., useful) means of extracting and summarising game information. Although the use of the WDA approach in the present study was somewhat limited by the small number of SME’s who participated in the study, the SME’s involved had extensive experience across several continents and in major tournaments as coaches, players, and match analysts. Despite the substantial use of the WDA in other research topics, further validation of this method is needed for PA in football. For example the current method could be applied using SME’s with different cultural demographics to those of the current study.

Recent comments from researchers have indicated the need for a multi-disciplinary approach for the development of sport science [1, 3, 9]. Applying HF methods to football research helps to address this issue, and can be used to examine the complexity of football and some of the gaps in existing PA methods. Furthermore, we have identified that a research-practitioner gap exists that needs to be addressed in order for PA research to be beneficial in everyday practice for coaches and practitioners. Lastly, several new PA functions and measures have been identified, which in the opinion of football experts, has the potential to advance our understanding of PA in football.

## Conclusion

The current study indicated that a different approach is needed to advance the current approaches used in PA for football. In particular, the novel measures identified in the current study require new measurement techniques, and the complexity engendered during football matches requires an integrated approach that considers multiple aspects of performance. The challenge for researchers is to develop and test these new measures to move PA research forward and to better align PA with the needs of coaches. In our opinion, to align research and practice more closely, the integration of sport scientists and football experts is required to fully understand PA in football. It appears that current PA measures are driven by researcher-based approaches that are largely impractical and unusable in practice. Through demonstrating that football is a complex system that requires new ideas and potentially more sophisticated, yet useable, measurement techniques, we hope that this article provides the impetus to bridge this research-practice gap.
